# Supplementary material for: β-arrestin1/YAP/mutant p53 complexes orchestrate the endothelin A receptor signaling in high-grade serous ovarian cancer
Source: Nat Commun. 2019 Jul 19;10:3196. doi: 10.1038/s41467-019-11045-8 (PMC6642155; doi:10.1038/s41467-019-11045-8)
Supplement: Supplementary file 1 — Supplementary Information [file 41467_2019_11045_MOESM1_ESM.pdf]

Supplementary Information

β-arrestin1/YAP/mutant p53 complex orchestrates the endothelin A receptor signaling in high-grade serous ovarian cancer

Tocci et al.

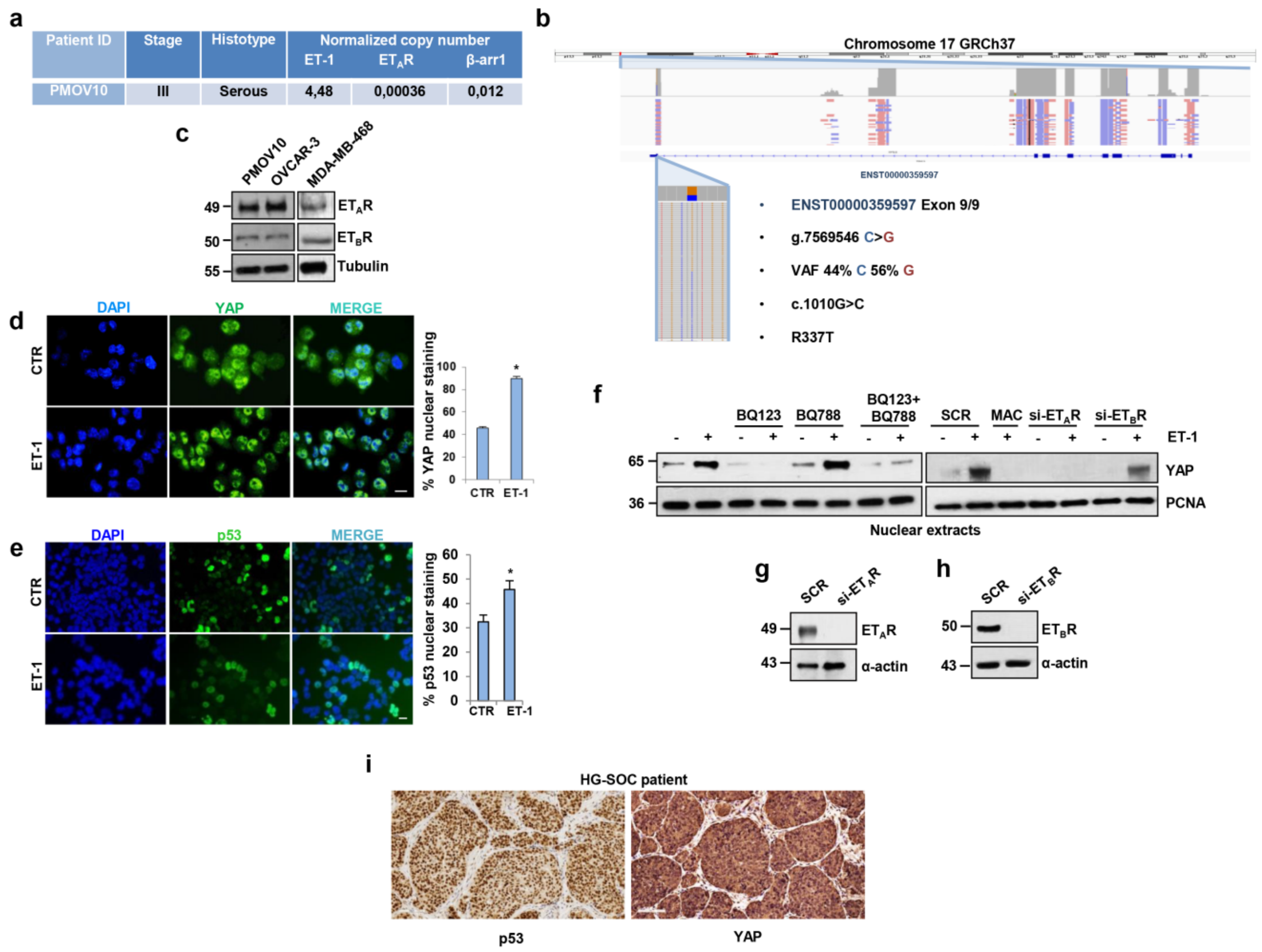

Supplementary Fig. 1 Characterization of HG-SOC patient-derived cell line

a PMOV10 cells isolated by the ascitic fluid of HG-SOC patient (III stage), were characterized by qRT-PCR for ET-1, ET$_A$R and β-arr1 mRNA levels expressed as copy number. **b** *TP53* gene sequencing and IGV visualization of single nucleotide (C>G) missense mutation variant (R337T) on the exon 9 in PMOV10 cells. The observed genomic nucleotide variant is a C to G, on chromosome 17, position 7569546 (hg19). **c** Immunoblotting (IB) analysis of ET$_A$R and ET$_B$R protein expression in total extracts of patient-derived HG-SOC cells (PMOV10), HG-SOC cell line (OVCAR-3), and breast cancer cell line (MD-MB-468), carrying TP53 mutations. Tubulin was used as loading control. **d, e** IF analysis of YAP (d) (scale bar: 20 µm, magnification 100X) and p53 (e) (scale bar: 20 µm, magnification 40X) in PMOV10 cells stimulated or not with ET-1 (100 nM) for 90 min. Nuclei are stained in blue (DAPI). *Right graphs* represent the percentage of YAP and p53 nuclear staining. Bars are means ± SD (*, p<0.001 vs CTR). **f** IB analysis of YAP protein levels in

nuclear extracts of PMOV10 cells treated with the selective ET$_A$R antagonist BQ123 (1μM), or with the ET$_B$R antagonist BQ788 (1μM), or with both BQ123+ BQ788, or with MAC, or silenced for ET$_A$R or ET$_B$R for 72 h, and stimulated or not with ET-1 for 90 min. PCNA was used as loading control. **g, h** PMOV10 cells transfected with si-ET$_A$R (g) or si-ET$_B$R (h) for 72 h were IB for ET$_A$R and ET$_B$R, respectively. α-actin was used as loading control. **i** p53 and YAP staining by IHC analysis in the HG-SOC patient from which PMOV10 cells were derived (scale bar: 100 μm, magnification 200X).

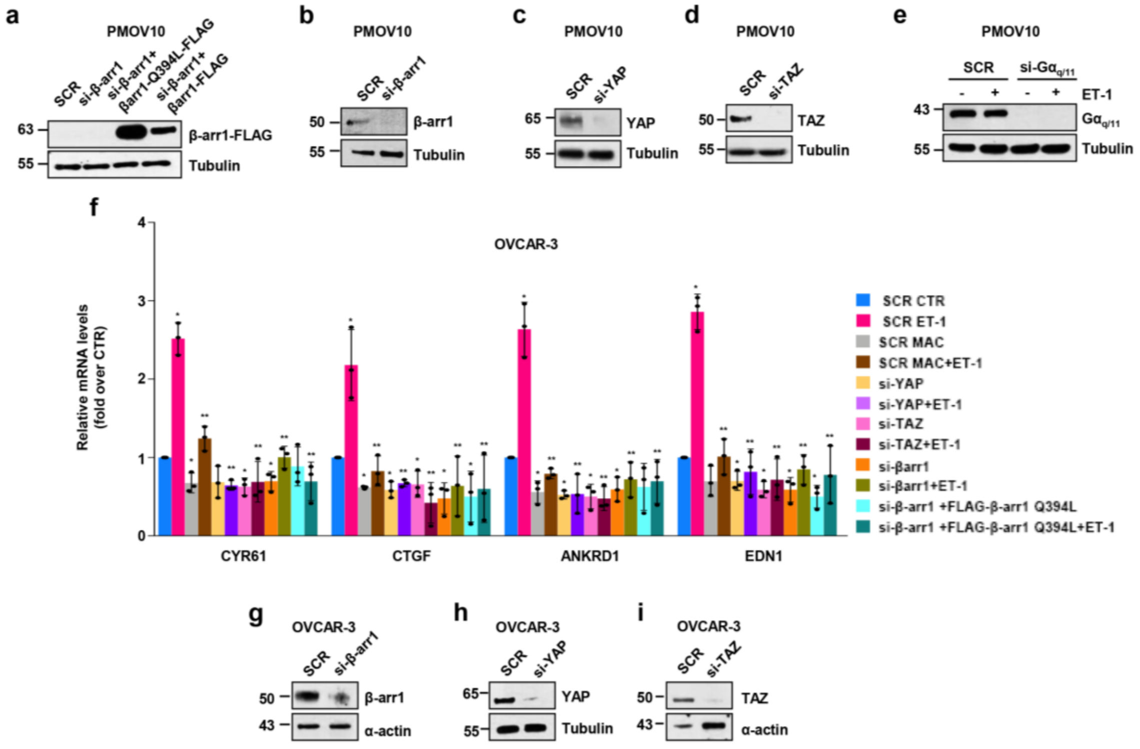

Supplementary Fig. 2 β-arr1 interacts with YAP mediating target gene expression

a Total extracts of PMOV10 cells transfected with SCR, or si-β-arr1, or si-β-arr1 and mutant β-arr1Q394L-FLAG, unable of nuclear localization, an then rescued with β-arr1-FLAG were IB for β-arr1. Tubulin was used as loading control. **b-d** PMOV10 cells transfected with si-β-arr1 (c) or si-YAP (d) or si-TAZ (d) for 72 h were IB for β-arr1, or YAP, or TAZ, respectively. Tubulin was used as loading control. **e** PMOV10 cells transfected with si-GNAQ (G$\alpha_{q/11}$) for 72 h and treated with

ET-1 for 90 min were IB for Gαq/11. Tubulin was used as loading control. **f** Expression analysis (qRT-PCR) of the indicated YAP target genes in OVCAR-3 cells stimulated with ET-1 (100 nM) and/or MAC (1 µM) for 24 h or transfected with si-YAP, or si-TAZ, or si-β-arr1, si-β-arr1 and mutant β-arr1Q394L-FLAG. Bars are means ± SD (*, p<0.01 vs CTR; **, p<0.01 vs ET-1) (n=3). **g-i** OVCAR-3 cells transfected with si-β-arr1 (g) or si-YAP (h) or si-TAZ (i) for 72 h were IB for β-arr1, or YAP, or TAZ, respectively. Tubulin or α-actin was used as loading control.

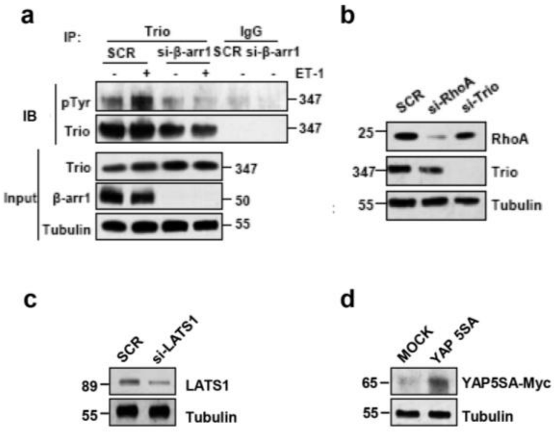

Supplementary Fig. 3 ET-1R/β-arr1 mediates RhoA signalling and YAP nuclear accumulation through Trio and LATS

a Total extracts of PMOV10 cells silenced or not for β-arr1 for 72 h and stimulated with ET-1 (100 nM) for 5 min were IP for endogenous Trio by using anti-Trio or anti-IgG and IB using anti-Trio and anti-pTyr Abs. Tubulin was used as loading control. **b-d** PMOV10 cells transfected with si-RhoA or si-Trio (b) or si-LATS (c) or YAP5SA (d) for 72 h were analyzed by IB for indicated proteins. Tubulin was used as loading control.

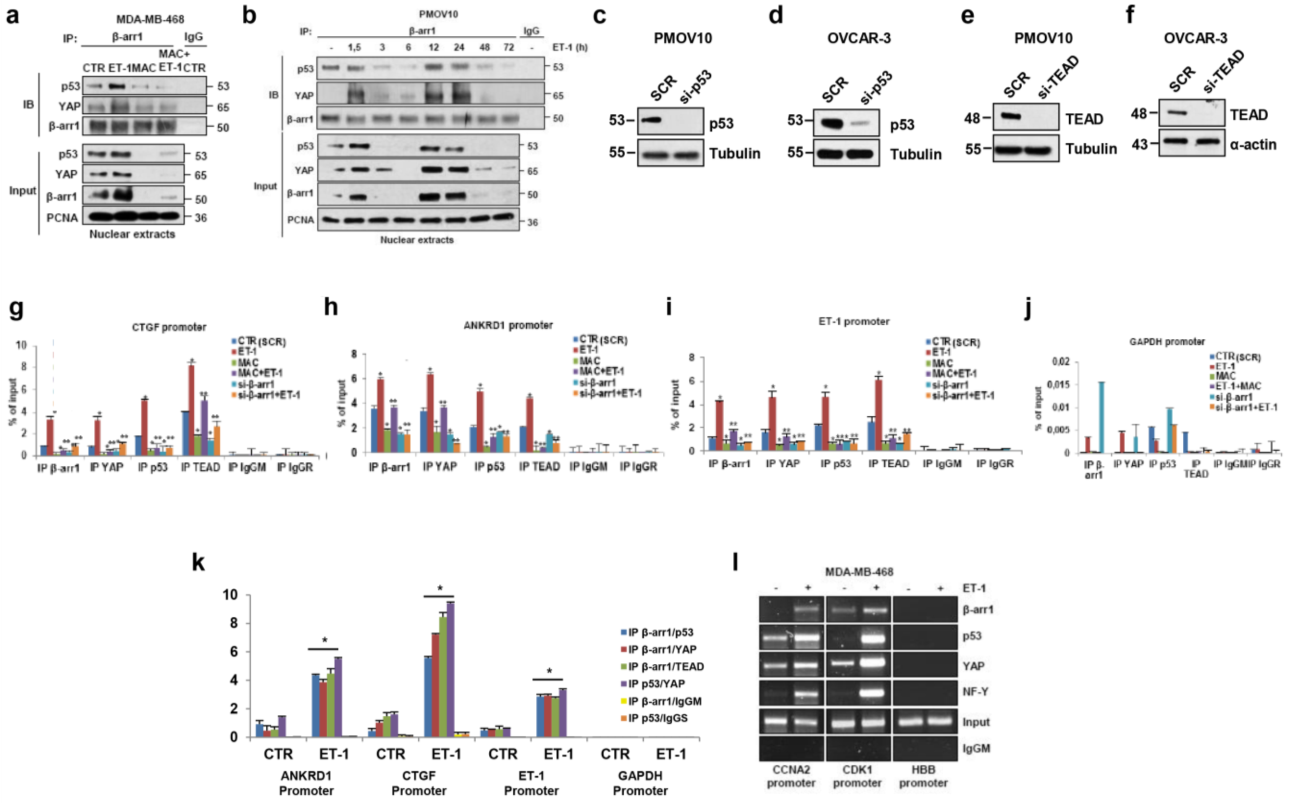

Supplementary Fig. 4 β-arr1 and mutp53 form a nuclear complex with YAP and TEAD or NFY to mediate ET-1R-induced transcriptional program

a Nuclear extracts of MDA-MB-468 cells stimulated with ET-1 and/or MAC for 90 min were IP for endogenous β-arr1 using anti-β-arr1, or anti-IgG Abs and IB using anti-β-arr1, anti-p53 and anti-YAP. PCNA was used as nuclear loading control. **b** PMOV10 cells stimulated with ET-1 for different time points were IP for endogenous β-arr1 using anti-β-arr1, or anti-IgG Abs and IB using anti-β-arr1, anti-p53 and anti-YAP.PCNA was used as nuclear loading control. **c-f** PMOV10 (c, e) or OVCAR-3 (d, f) cells transfected with si-p53 (c, d) or si-TEAD (e, f) for 72 h were IB for p53, or TEAD, respectively. Tubulin or α-actin were used as loading control. **g-j** PMOV10 cells were transiently transfected with SCR or si-β-arr1 for 72 h and stimulated with ET-1 (100 nM) and/or MAC (1 μM) for 24 h. The binding of β-arr1, YAP, p53 and TEAD on *CTGF* promoter (g), *ANKRD1* promoter (h), *ET-1* promoter (i) and *GAPDH* promoter (j), used as negative control, was measured by ChIP analysis followed by qRT-PCR. Non-specific anti-IgG mouse or rabbit Abs

(IgGM, IgGR) were used as control for all ChIP reactions. Bars are means ± SD (*, p<0.001vs CTR; **, p<0.001vs ET-1) (n=3). **k** The co-occupancy of β-arr1/YAP/TEAD/p53 to the promoters of *ANKRD1*, *CTGF*, *ET-1* and *GAPDH*, used as negative control, was measured in PMOV10 cells stimulated or not with ET-1 (100 nM) for 24 h by ChIP-re-ChIP assays followed by qRT-PCR. Non-specific anti-IgG mouse or sheep Abs (IgGM, IgGS) were used as control for all ChIP reactions. Bars are means ± SD (*, p<0.01 vs CTR). **l** MDA-MB-468 cells were stimulated with ET-1 for 90 min. The binding of β-arr1, YAP, TEAD, p53, and NFY on the promoters of *CCNA2*, *CDK1*. *HBB* (Haemoglobin, negative control locus) was measured by ChIP analysis followed by PCR. Anti-IgG mouse or rabbit Abs (IgGM) were used as control for all ChIP reactions.

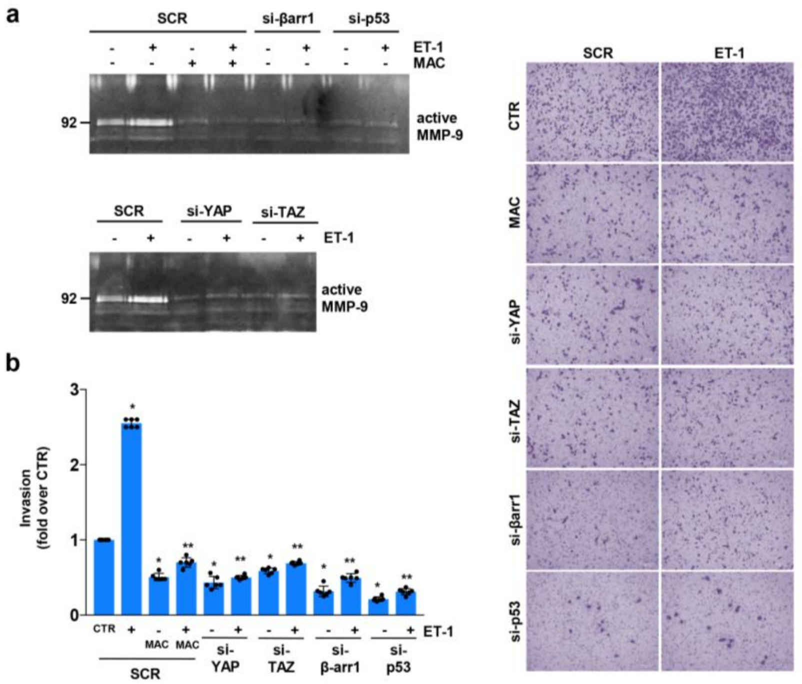

Supplementary Fig. 5 ET-1R/β-arr1 axis promotes HG-SOC invasion through the formation of mutant p53/YAP complex

a PMOV10 cells treated with ET-1 (100 nM) and/or MAC (1 μM) for 24 h were transfected with SCR, or si-β-arr1, or si-p53, or si-YAP, or si-TAZ for 72 h. Cultured media were collected and analyzed by gelatin zymography to visualize active MMP-9. **b** Invasion assays of PMOV10 cells

transfected with SCR, or si-YAP, or si-TAZ, or si-β-arr1, or si-p53 and treated with ET-1 (100 nM) and/or MAC (1µM) for 24 h. Bars are means ± SD (*, p<0.02 vs CTR; **, p<0.001 vs SCR ET-1). The invasive cells are counted (*Left*) or photographed (*Right panels*, Magnification X4).

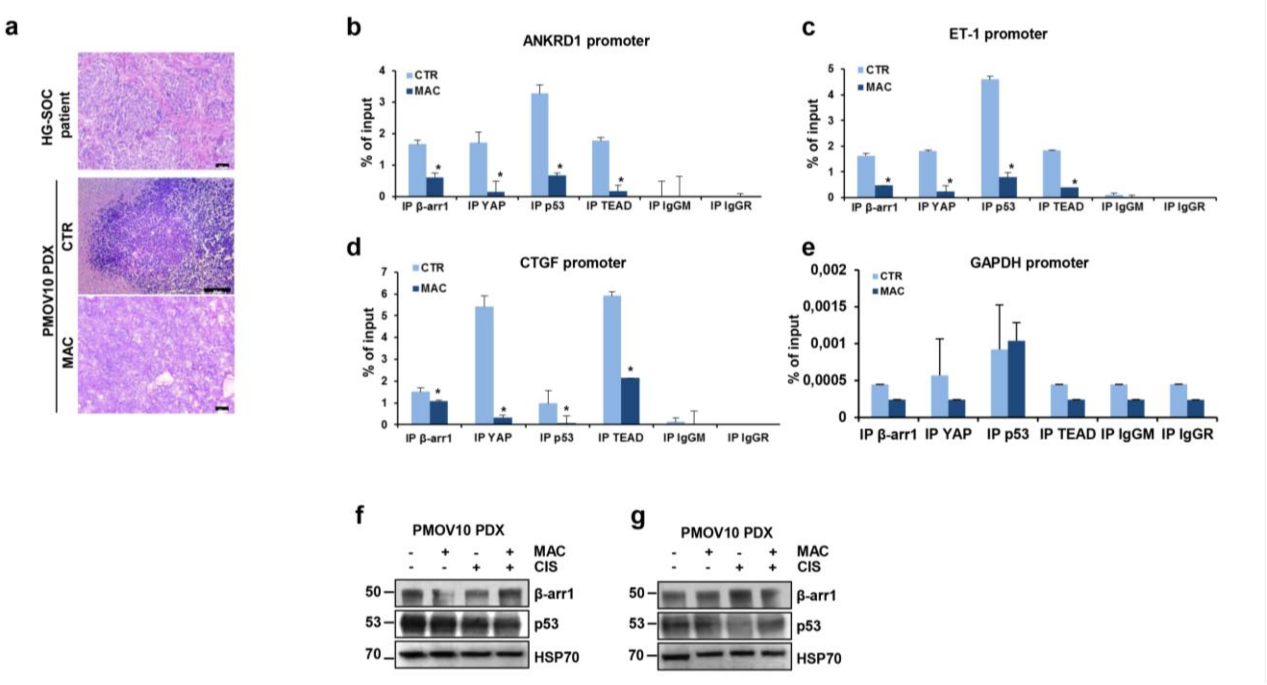

Supplementary Fig. 6 ET-1R blockade by macitentan, interferes with YAP/mutp53/TEAD transcriptional activity in HG-SOC PDX

a Hematoxylin-eosin (H&E) staining of HG-SOC patient section from which PMOV10 cells were derived and of sections of tumor PMOV10 PDX treated or not with MAC (scale bar: 50 µm, magnification 20X). **b-e** The binding of β-arr1, YAP, TEAD and p53 to the promoters of *ANKRD1* (b), *ET-1* (c), *CTGF* (d) and *GAPDH* (e), used as negative control, was analyzed in PMOV10 PDX treated or not with MAC (1µM) by ChIP assays followed by qRT-PCR. Non-specific anti-IgG mouse or rabbit Abs (IgGM, IgGR) were used as control for all ChIP reactions. Bars are means ± SD (*, p<0.001 vs CTR) (n=3). **f, g** β-arr1 and p53 protein expression was evaluated by IB analysis of subcutaneously tumors (f) or intraperitoneal nodules (g) extracts of PMOV10 PDX treated with MAC and/or CIS and MAC+CIS. HSP70 was used as loading control

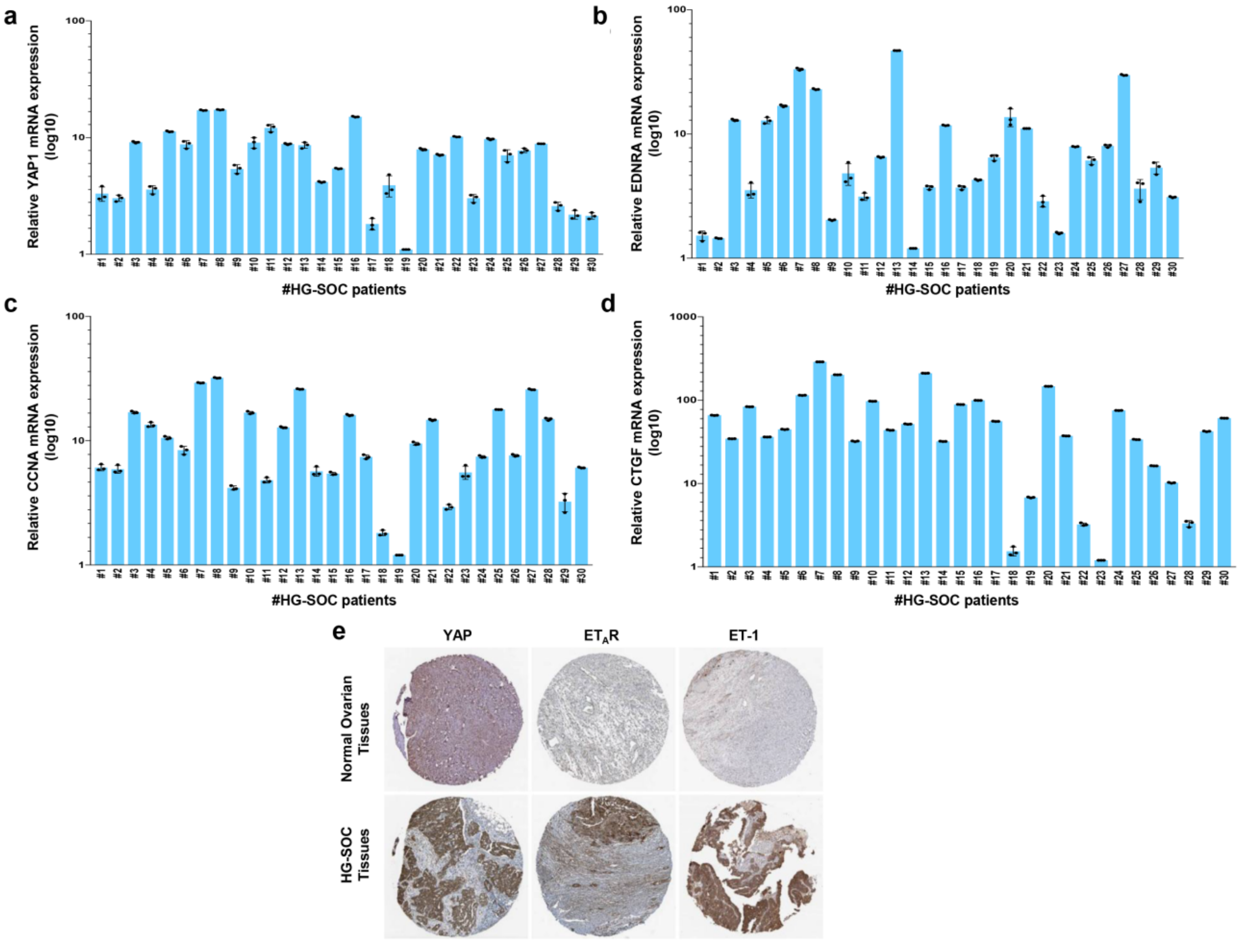

Supplementary Fig. 7 Expression of ET-1, ET$_A$R and YAP in HG-SOC patients

a-d Relative *YAP1* (a), *EDNRA* (ET$_A$R) (b), *CCNA* (cyclin A) (c) and *CTGF* (d) mRNA expression levels by qRT-PCR in 30 HG-SOC human specimens normalized for *CYPA* mRNA expression. Bars are means ±SD. **e** YAP, ET$_A$R and ET-1 expression in normal ovarian tissue and HG-SOC specimens. Images were taken from the Human Protein Atlas (http://www.proteinatlas.org) database.

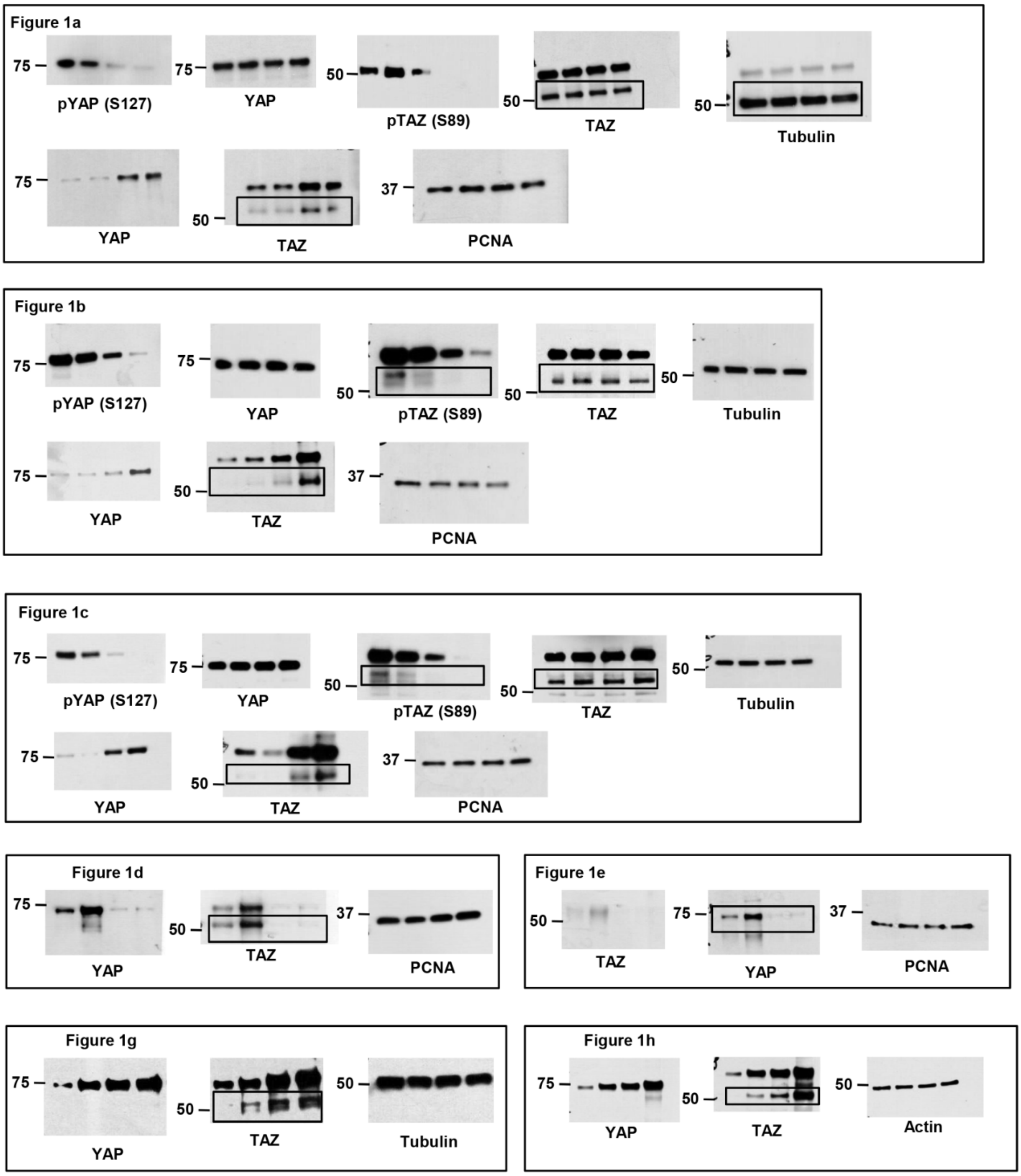

Supplementary Fig. 8. Uncropped western blots of Fig. 1

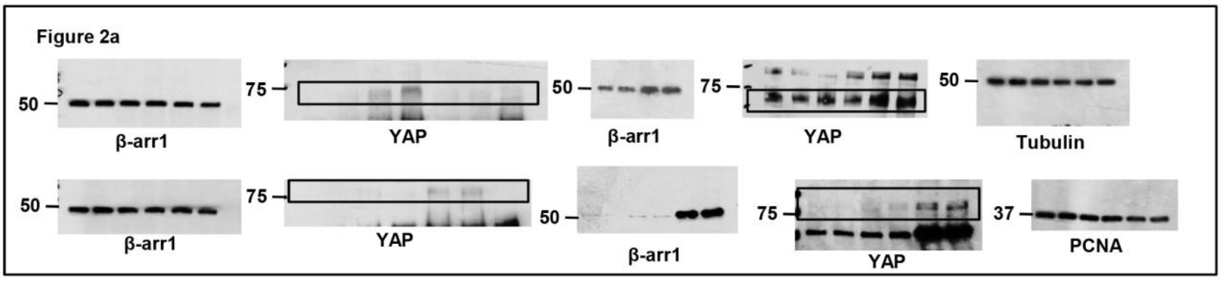

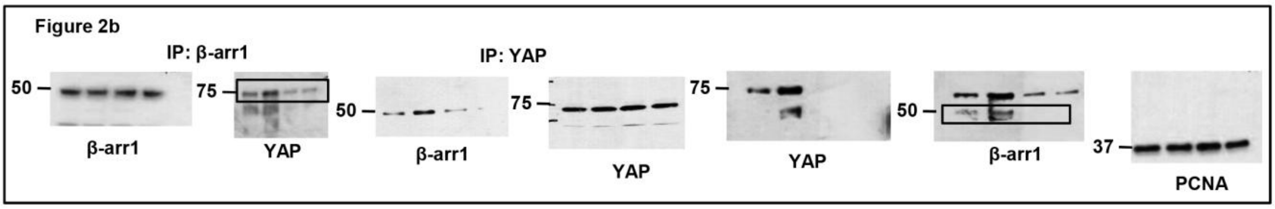

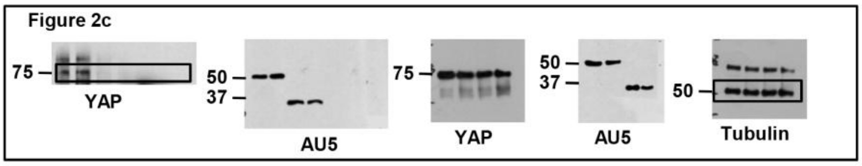

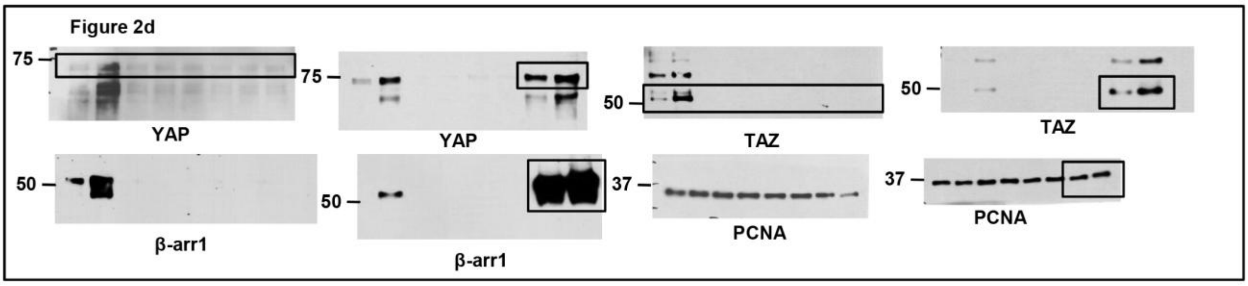

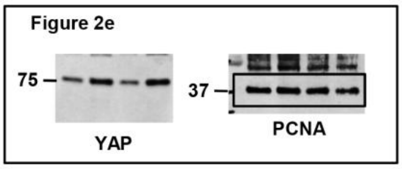

Supplementary Fig. 9. Uncropped western blots of Fig. 2

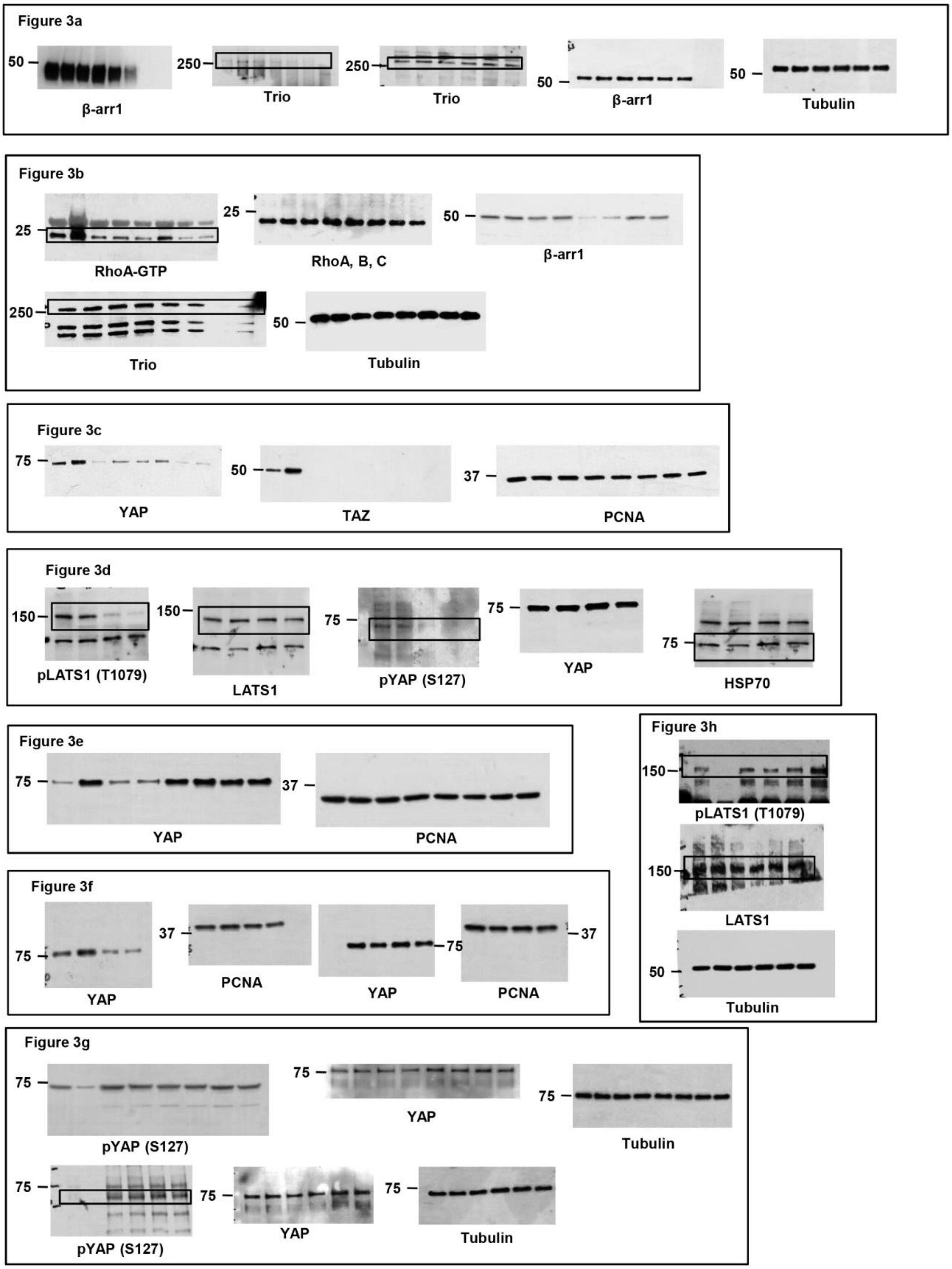

Supplementary Fig. 10. Uncropped western blots of Fig. 3

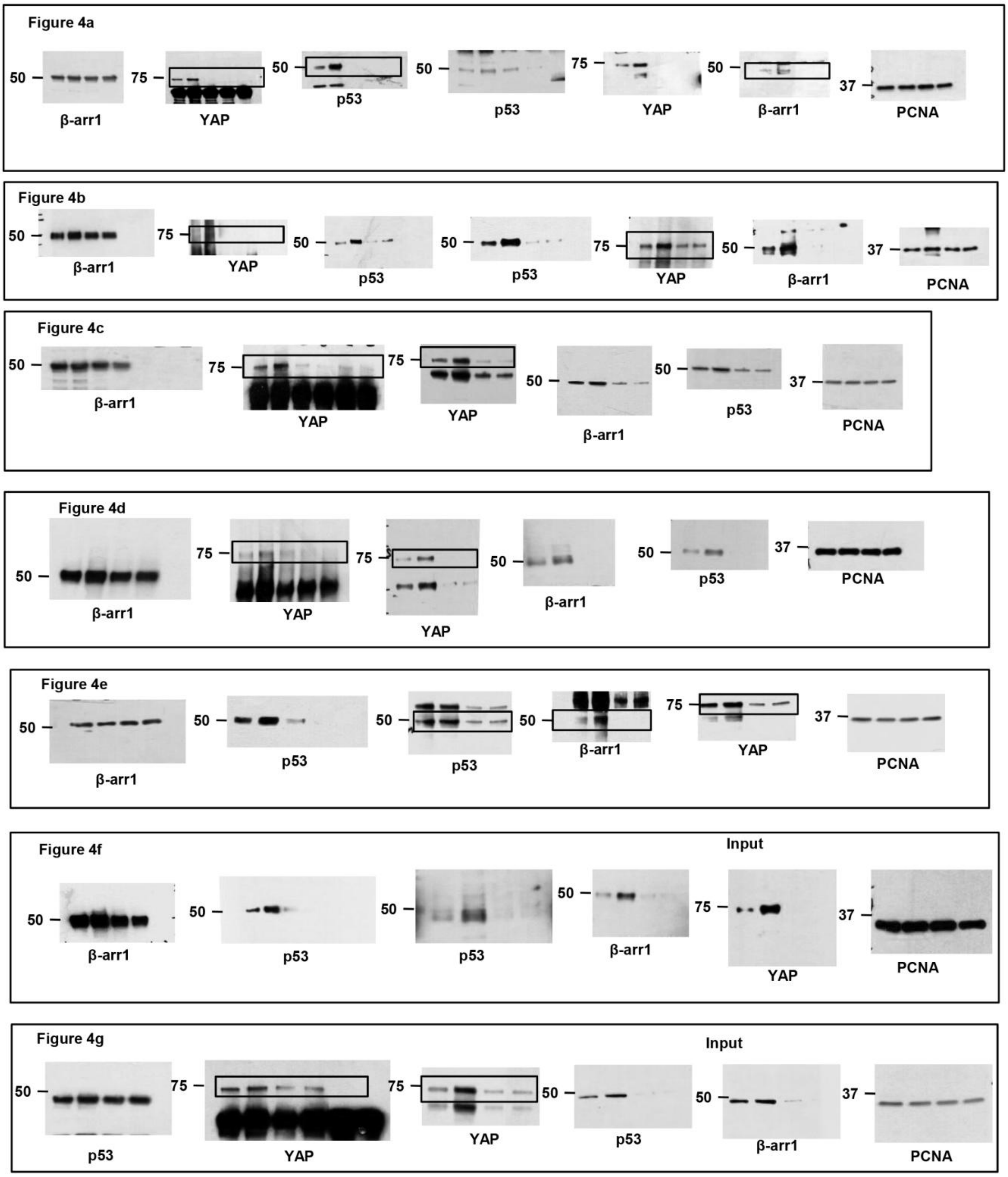

Supplementary Fig. 11. Uncropped western blots of Fig. 4

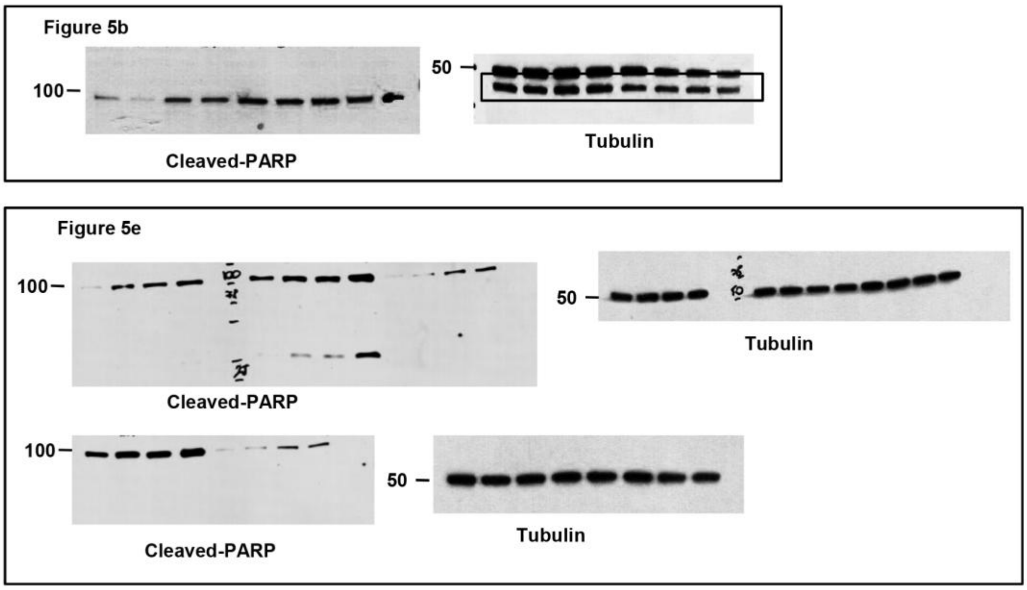

Supplementary Fig. 12A. Uncropped western blots of Fig. 5

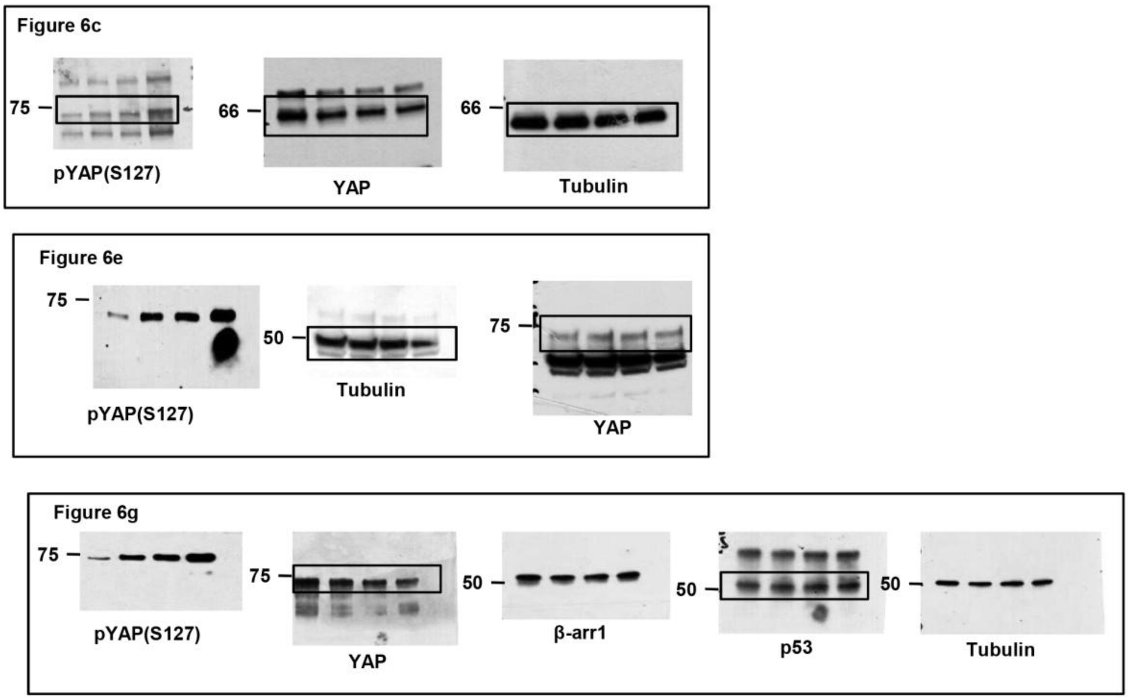

Supplementary Fig. 12B. Uncropped western blots of Fig. 6

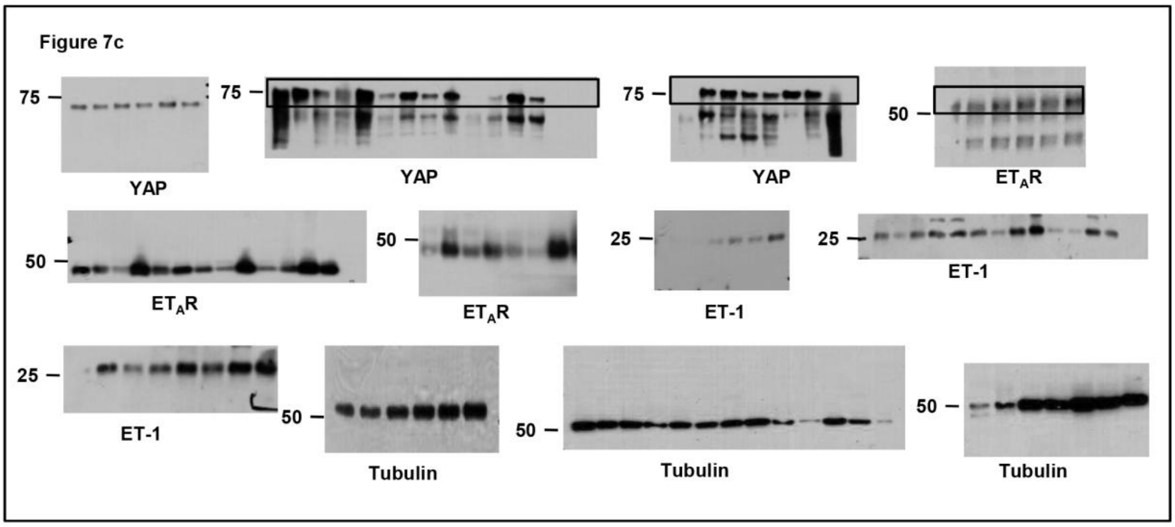

Supplementary Fig. 13A. Uncropped western blots of Fig. 7

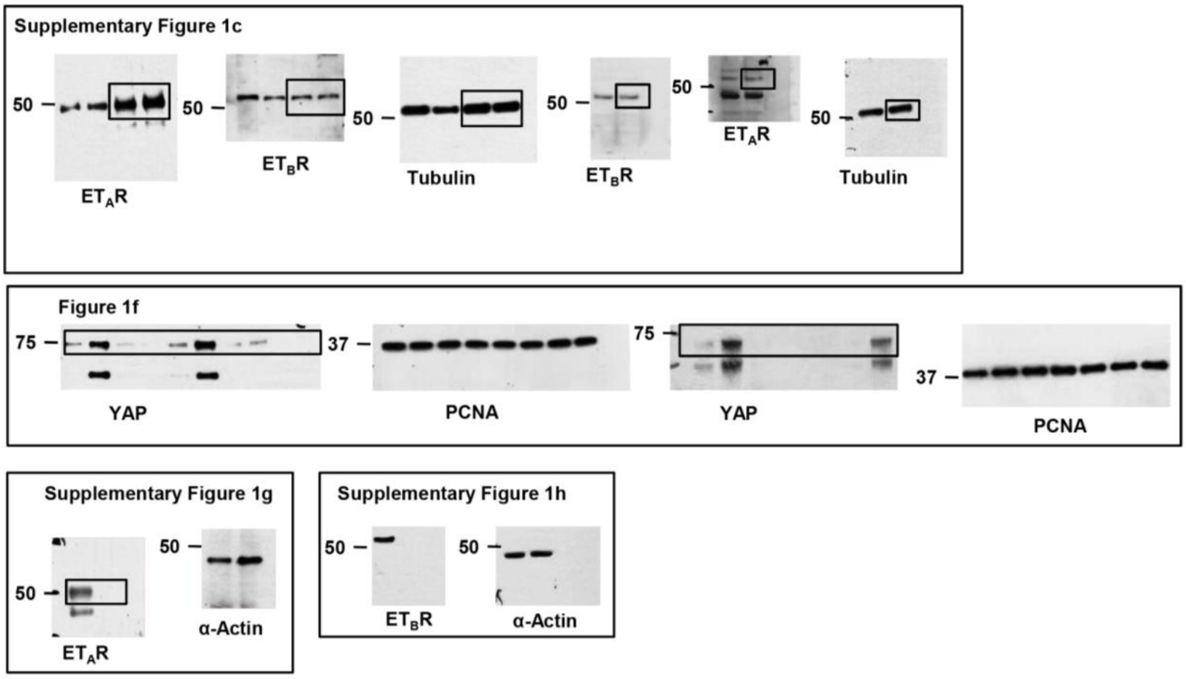

Supplementary Fig. 13B. Uncropped western blots of Supplementary Fig. 1

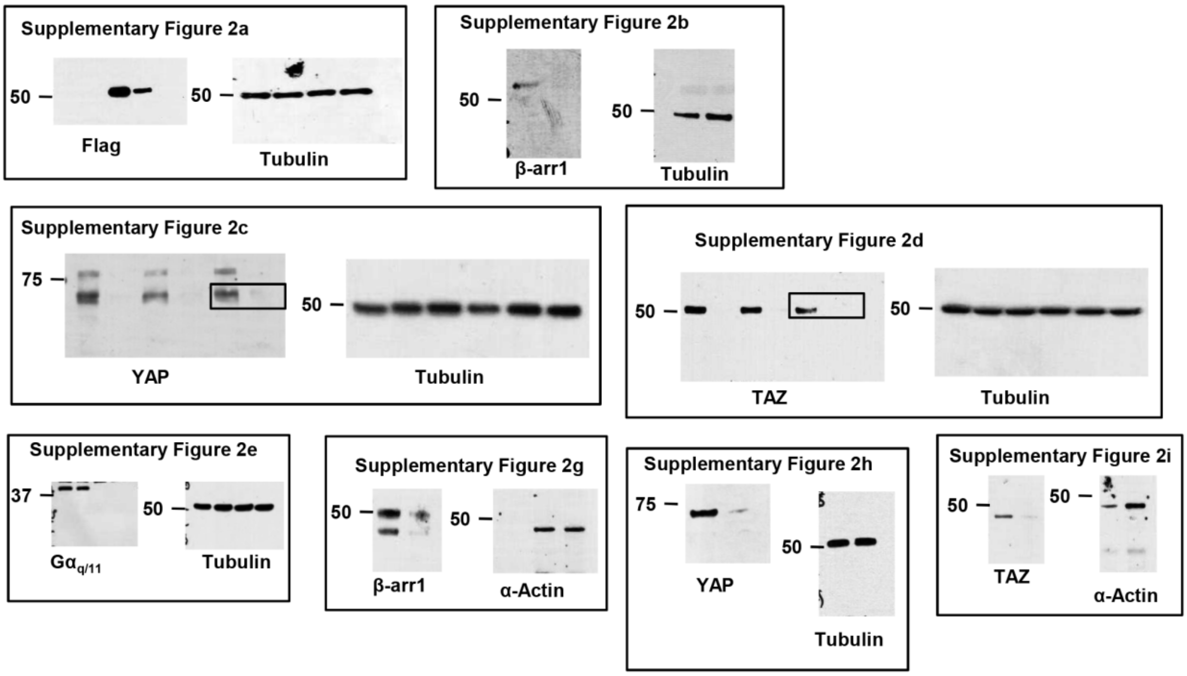

Supplementary Fig. 14A. Uncropped western blots of Supplementary Fig. 2

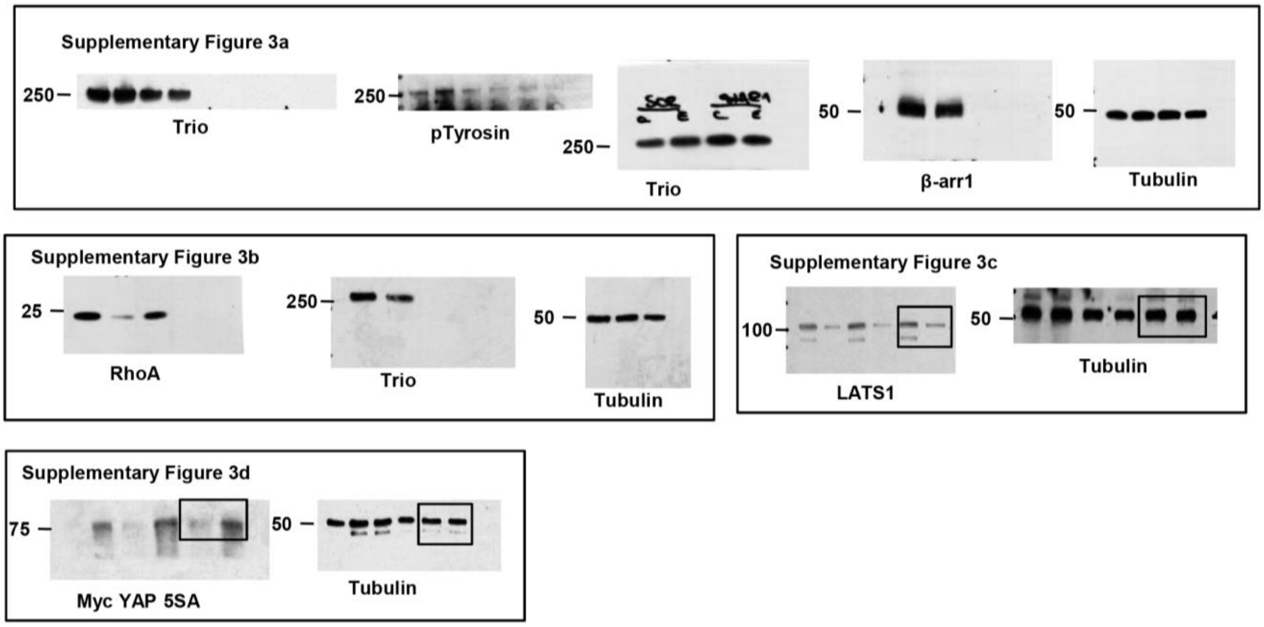

Supplementary Fig.14 B. Uncropped western blots of Supplementary Fig. 3

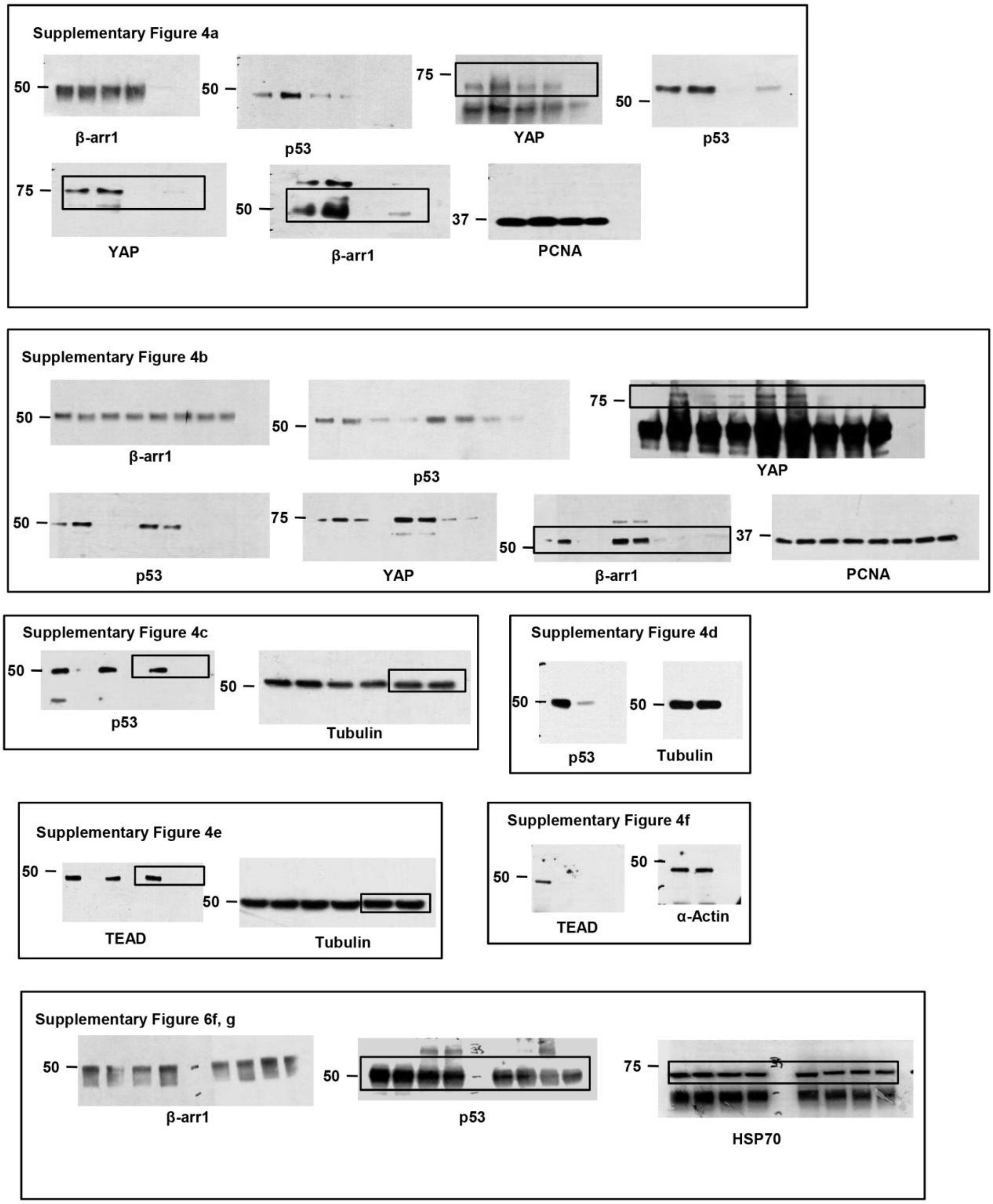

Supplementary Fig.15. Uncropped western blots of Supplementary Fig. 4 and 6

Supplementary Table 1. Antibodies used for Immunoblotting		
**Antigen**	**Diluition**	**Manufacturer**
PCNA (F-2) sc-25280	1:200	Santa Cruz Biotechology
Tubulin (DM1A) sc-32293	1:201	Santa Cruz Biotechology
ETAR ab84673	1:1000	Abcam
ETBR ab39960	1:1000	Abcam
pYAP (S127) D9W2I cat no. 13008	1:1000	Cell Signaling
YAP 1A12 cat. no. 12395	1:1000	Cell Signaling
pTAZ (S89) E1X9C cat. no. 59971	1:1000	Cell Signaling
TAZ cat. no. HPA007415	1:250	Sigma Aldrich
β-arrestin 1 E274 ab32099	1:500	Abcam
FLAG cat. no. 2368	1:1000	Cell Signaling
AU5 ab24576	1:500	Abcam
Trio (H-120) sc-28564	1:200	Santa Cruz Biotechology
pTyrosincat. no. 9411	1:2000	Cell Signaling
RhoA-GTP	1:1000	Cytoskeleton, Inc.
RhoA, B, C clone 55 cat. no. 05-778	1:500	Millipore
pLATS1 (T1079) D57D3 cat. no. 8654	1:1000	Cell Signaling
LATS1 cat. no. A300-477A	1:5000	Bethyl
Myc 9E10 cat. no. TA150121	1:500	OriGene
p53 (DO1) sc-126	1:200	Santa Cruz Biotechology
Cleaved-PARP cat. no. 9541	1:1000	Cell Signaling
GNAQ ab75825	1:1000	Abcam
TEAD (TEF-3, N-G2) sc-101184	1:200	Santa Cruz Biotechology
α-actin (1A4) sc-32251	1:200	Santa Cruz Biotechology
HSP70 (3A3) sc-32239	1:200	Santa Cruz Biotechology

Supplementary Table 2: Primer List

Primers used for RT-qPCR experiments

Target gene	Primer sequences
CYR61 F	5’-CAGGACTGTGAAGATGCGGT-3’
CYR61 R	5’-GCCTGTAGAAGGGAAACGCT-3’
CTGF F	5’-AGGAGTGGGTGTGTGACGA-3’
CTGF R	5’-CCAGGCAGTTGGCTCTAATC-3’
ANKRD1 F	5’-AGTAGAGGAACTGGTCACTGG-3’
ANKRD1 R	5’-TGGGCTAGAAGTGTCTTCAGA-3’
EDN1 F	5’-CCAAGAGAGCCTTGGAGAAT-3’
EDN1 R	5’-TGTCTTCAGCCCTGAGTTCTT-3’
EDNRA F	5’-GGGATCACCGTCCTCAACCT-3’
EDNRA R	5’-CAGGAATGGCCAGGATAAAGG-3’
ARRB1 F	5’-GTGAAAGTGAAGCTGGTGGTGTC-3’
ARRB1 R	5’-TTCATGCCTTTCAGCCGCTG-3’
YAP F	5’-GCAAATTCTCCAAAATGTCAGG-3’
YAP R	5’-CGGGAGAAGACACTGGATTT-3’
CYCLOPHILIN-A F	5’-TTCATCTGCACTGCCAAGAC-3’
CYCLOPHILIN-A R	5’-TCGAGTTGTCCACAGTCAGC-3’
Cdk1 F	5'-GGAAGGGGTTCCTAGTACTGC-3'
Cdk1 R	5'-TGGAATCCTGCATAAGCACA-3'
CCNA2 F	5'-CCATACCTCAAGTATTTGCCATCA-3'
CCNA2 R	5'-AGCTTTGTCCCGTGACTGTGT-3'

Primers used in ChIP experiments

Promoter	Primer sequences
ET-1 F	5′-CAGCTTGCAAAGGGGAAGCG-3′
ET-1 R	5′- TCCGACTTTATTCCAGCCCC -3′
CTGF F	5’-CAATCCGGTGTGAGTTGATG-3’
CTGF R	5’-GCCAATGAGCTGAATGGAGT-3’
ANKRD1 F	5'-GAGGGGAGGACAAGCTAACC-3’
ANKRD1 R	5’-CGATGTGATCACCACCAAAG-3’
GAPDH F	5’-CGGGATTGTCTGCCCTAATTAT-3’
GAPDH R	5’-GCACGGAAGGTCACGATGT-3’.
CCNA2 F	5'-GAGTCAGCCTTCGGACAGCC-3'
CCNA2 R	5'-CCAGAGATGCAGCGAGCAGC-3'
CDK1 F	5'-GAACTGTGCCAATGCTGGGA-3'
CDK1 R	5'-GCAGTTTCAAACTCACCGCG-3'
HBB F	5'-GCTTCTGACACAACTGTGTTCACTAGC-3'
HBB R	5'-CACCAACTTCATCCACGTTCACC-3'
