# Supplementary material for: β-arrestin1/YAP/mutant p53 complexes orchestrate the endothelin A receptor signaling in high-grade serous ovarian cancer
Source: Nat Commun. 2019 Jul 19;10:3196. doi: 10.1038/s41467-019-11045-8 (PMC6642155; doi:10.1038/s41467-019-11045-8)
Supplement: Supplementary file 3 — Reporting Summary [file 41467_2019_11045_MOESM3_ESM.pdf]

nature research

Corresponding author(s): Anna Bagnato
Last updated by author(s): 2019-June- 6

Reporting Summary

Nature Research wishes to improve the reproducibility of the work that we publish. This form provides structure for consistency and transparency in reporting. For further information on Nature Research policies, see Authors & Referees and the Editorial Policy Checklist.

Statistics

For all statistical analyses, confirm that the following items are present in the figure legend, table legend, main text, or Methods section.

n/a	Confirmed	
☐	☒	The exact sample size (*n*) for each experimental group/condition, given as a discrete number and unit of measurement
☐	☒	A statement on whether measurements were taken from distinct samples or whether the same sample was measured repeatedly
☐	☒	The statistical test(s) used AND whether they are one- or two-sided<br>*Only common tests should be described solely by name; describe more complex techniques in the Methods section.*
☒	☐	A description of all covariates tested
☐	☒	A description of any assumptions or corrections, such as tests of normality and adjustment for multiple comparisons
☐	☒	A full description of the statistical parameters including central tendency (e.g. means) or other basic estimates (e.g. regression coefficient) AND variation (e.g. standard deviation) or associated estimates of uncertainty (e.g. confidence intervals)
☒	☐	For null hypothesis testing, the test statistic (e.g. *F*, *t*, *r*) with confidence intervals, effect sizes, degrees of freedom and *P* value noted<br>*Give P values as exact values whenever suitable.*
☒	☐	For Bayesian analysis, information on the choice of priors and Markov chain Monte Carlo settings
☒	☐	For hierarchical and complex designs, identification of the appropriate level for tests and full reporting of outcomes
☒	☐	Estimates of effect sizes (e.g. Cohen's *d*, Pearson's *r*), indicating how they were calculated

Our web collection on statistics for biologists contains articles on many of the points above.

Software and code

Policy information about availability of computer code

Data collection	No software code was used for data collection
Data analysis	Statistical analyses were performed with GraphPad Prism 7. Gray values for Western blotting were analyzed using Image J.

For manuscripts utilizing custom algorithms or software that are central to the research but not yet described in published literature, software must be made available to editors/reviewers. We strongly encourage code deposition in a community repository (e.g. GitHub). See the Nature Research guidelines for submitting code & software for further information.

Data

Policy information about availability of data

All manuscripts must include a data availability statement. This statement should provide the following information, where applicable:

- Accession codes, unique identifiers, or web links for publicly available datasets
- A list of figures that have associated raw data
- A description of any restrictions on data availability

All relevant data are available from the corrisponding author upon reasonable request. Uncropped blots of major figures are shown in Supplementary Figures 8-15.

Field-specific reporting

Please select the one below that is the best fit for your research. If you are not sure, read the appropriate sections before making your selection.

☒ Life sciences ☐ Behavioural & social sciences ☐ Ecological, evolutionary & environmental sciences

For a reference copy of the document with all sections, see nature.com/documents/nr-reporting-summary-flat.pdf

Life sciences study design

All studies must disclose on these points even when the disclosure is negative.

Sample size	10 mice per group were used in the mouse xenograft models study.
Data exclusions	No data were excluded from the analysis.
Replication	All attempts to replicate the experimental findings were successful.
Randomization	The allocation of cells/mice to different tretments was completely random.
Blinding	Immunohistochemical staining analysis of patient tissues was blinded.

Reporting for specific materials, systems and methods

We require information from authors about some types of materials, experimental systems and methods used in many studies. Here, indicate whether each material, system or method listed is relevant to your study. If you are not sure if a list item applies to your research, read the appropriate section before selecting a response.

Materials & experimental systems

n/a	Involved in the study	
☐	☒	Antibodies
☐	☒	Eukaryotic cell lines
☒	☐	Palaeontology
☐	☒	Animals and other organisms
☐	☒	Human research participants
☒	☐	Clinical data

Methods

n/a	Involved in the study	
☒	☐	ChIP-seq
☒	☐	Flow cytometry
☒	☐	MRI-based neuroimaging

Antibodies

Antibodies used	The antibodies used in this study are listed in Supplementary Table 1.
Validation	All antibodies that are commercially available have been tested for species reactivity and application by the manufacturers.

Eukaryotic cell lines

Policy information about cell lines

Cell line source(s)	OVCAR3 (HTB-161) and MDA-MB-468 (HTB-132) were obtained from American Type Culture Collection
Authentication	Cell line authentication was performed by short tandem repeat (STR)-profiling
Mycoplasma contamination	All cell lines were tested negative for mycoplasma contamination.
Commonly misidentified lines (See ICLAC register)	No misidentified cell lines were used in this study.

Animals and other organisms

Policy information about studies involving animals; ARRIVE guidelines recommended for reporting animal research

Laboratory animals	Female nude mice of 6-week old were from Charles River Lab., Milan, Italy
Wild animals	NA
Field-collected samples	This study did not involve samples collected from the field
Ethics oversight	All the animal experiments were performed in accordance with the Italian Ministry of Health guidelines and protocols after approval by the Animal Welfare Body of Regina Elena Cancer Institute of Rome.

Note that full information on the approval of the study protocol must also be provided in the manuscript.

Human research participants

Policy information about studies involving human research participants

Population characteristics	All patients analyzed in this study were diagnosed with high-grade serous ovarian cancer between 2016 and 2017 at the Gynecologic Oncology Unit at the Catholic University of Rome.
Recruitment	The tumor samples were primary tumours collected for diagnosis before treatment. These archival tissue blocks were with confirmed diagnosis of high-grade serous ovarian cancer. The inclusion of tumor tissues was random.
Ethics oversight	Institutional Review Board of the Catholic University of Rome and Regina Elena Cancer Institute of Rome.

Note that full information on the approval of the study protocol must also be provided in the manuscript.
